# Serum Thyroid-Stimulating Hormone (sTSH) Levels as a Predictor of Thyroid Malignancy: A Retrospective Analysis of 102 Patients

**DOI:** 10.7759/cureus.81599

**Published:** 2025-04-02

**Authors:** Sandeepa D Dadigamuwage, Gayanga J Kottegoda, Dilini E Kannangara, Janani K Bhagya, S Suganthini, Subhashaba A Gunawardena

**Affiliations:** 1 Colorectal Surgery, University Hospitals Plymouth NHS Trust, Plymouth, GBR; 2 Surgery, Postgraduate Institute of Medicine, University of Colombo, Colombo, LKA; 3 Neurosurgery, Ministry of Health, Colombo, LKA; 4 Clinical Nutrition, Postgraduate Institute of Medicine, University of Colombo, Colombo, LKA; 5 General Surgery, Sri Jayewardenepura General Hospital, Colombo, LKA; 6 Critical Care, Colombo South Teaching Hospital, Colombo, LKA

**Keywords:** fine-needle aspiration cytology, papillary carcinoma of thyroid, thyroid nodule malignancy, thyroid-stimulating hormone (tsh), ultrasonography (usg) in thyroid nodules

## Abstract

Introduction

Thyroid nodules are common, with a significant proportion presenting with indeterminate cytology on fine-needle aspiration cytology (FNAC). Thyroid-stimulating hormone (TSH) has been proposed as a potential biochemical marker to aid in malignancy risk stratification, though findings across studies remain inconsistent. This study evaluates the association between preoperative serum TSH (sTSH) levels and histopathologically confirmed malignancy in patients undergoing thyroidectomy.

Method

We conducted a retrospective observational study on 102 patients who underwent total or partial thyroidectomy between 2019 and 2022 at a tertiary care centre. Preoperative sTSH levels, FNAC results, ultrasound characteristics, and final histopathological outcomes were analysed. Statistical analysis included independent t-tests and chi-squared tests to assess associations.

Results

Of the 102 patients, 18 (17.6%) were diagnosed with malignant lesions, predominantly papillary thyroid carcinoma. The mean sTSH level was slightly higher in malignant cases (1.378 μIU/mL) compared to benign cases (1.350 μIU/mL), but this was not statistically significant (p = 0.68). Histopathological features such as lymphovascular invasion (LVI) and extrathyroidal extension (ETE) were significantly more common in malignant cases (p < 0.01). FNAC had limited predictive accuracy, correctly identifying 61.1% of malignant cases.

Conclusion

Whilst no significant association was found between sTSH levels and malignancy, features such as LVI, ETE, and certain ultrasound characteristics remain valuable predictors. sTSH may be useful as part of a broader, multimodal risk assessment approach but should not be relied upon as a standalone diagnostic tool.

## Introduction

Thyroid nodules are frequently encountered in clinical practice, with an estimated prevalence of 19%-68% in the general population when detected via ultrasonography (USG). The risk of malignancy within these nodules varies from 5% to 15%, depending on factors such as iodine sufficiency, geographic location, and individual risk factors [1]. Currently, the gold standard for thyroid nodule evaluation includes USG, fine-needle aspiration cytology (FNAC), and molecular testing. However, FNAC results remain indeterminate in 15%-30% of cases, necessitating additional malignancy predictors [2].

Thyroid-stimulating hormone (TSH) is a crucial regulator of thyroid growth and function, acting through TSH receptor activation. Several studies have proposed a relationship between elevated TSH levels and increased thyroid cancer risk [3]. The hypothesis suggests that higher TSH levels stimulate thyrocyte proliferation, potentially contributing to neoplastic transformation. However, other studies have found no significant correlation between TSH and malignancy risk [4,5]. Given this conflicting evidence, further research is needed to determine whether preoperative TSH levels can reliably predict malignancy in thyroid nodules.

In clinical decision-making, especially when cytological results are inconclusive, having reliable biochemical or radiological adjuncts becomes invaluable. Incorporating serum biomarkers such as TSH could aid in risk stratification, helping to tailor the extent of surgical intervention and avoid unnecessary procedures in low-risk patients. Additionally, TSH testing is inexpensive, widely available, and routinely performed in preoperative assessment, making it a potentially accessible and cost-effective tool.

Understanding the biological behaviour of thyroid cancers and their association with hormonal influences could contribute to the refinement of existing guidelines. Identifying predictors of malignancy not only facilitates timely diagnosis but also supports appropriate clinical management, reducing patient anxiety and healthcare burden.

This study evaluates the association between preoperative serum TSH (sTSH) levels and histopathologically confirmed malignancy in a cohort of patients undergoing thyroidectomy. By analysing these patients, we aim to contribute to the ongoing debate regarding TSH as a potential predictor of malignancy and assess its clinical utility.

## Materials and methods

Methods

This study was designed as a retrospective observational cohort study and was conducted at Sri Jayewardenepura General Hospital (SJGH), a tertiary care teaching hospital in Sri Lanka. The study included patients who underwent total or partial thyroidectomy under the care of a single consultant-led surgical unit between January 2019 and December 2022. Ethical approval for the study was obtained from the Postgraduate Institute of Medicine, University of Colombo.

Study population

The inclusion criteria consisted of patients aged 18 years and older who presented to the surgical clinic at ward 08, SJGH, with anterior neck lumps and underwent thyroidectomy during the study period. Patients were excluded if they had a history of autoimmune thyroid disease, they were receiving thyroxine or anti-thyroid medication within one year before surgery, or key data such as preoperative sTSH levels or histological outcomes were unavailable.

Data collection

Data were collected from clinical records, operative notes, laboratory investigations, and histopathology reports. Where feasible, patients attending postoperative follow-up clinics were invited to complete an interviewer-administered questionnaire after providing informed written consent. This was used to obtain additional demographic or clinical data not available in the records.

Preoperative assessment

All patients underwent thyroid USG and FNAC prior to surgery. FNAC results were categorised using the Bethesda classification system [6]. USG was performed by consultant radiologists, and in cases where scans were conducted by trainees, images were reviewed and confirmed by supervising consultants. sTSH was measured using a standard chemiluminescent immunoassay with a reference range of 0.4-4.0 μIU/mL, and values were recorded within one month prior to surgery. In patients with multinodular goitre, the dominant nodule or the one with suspicious ultrasound features-such as irregular margins, hypoechogenicity, or microcalcifications-was selected for cytological sampling.

Surgical and histopathological evaluation

Thyroidectomies were performed by the consultant surgeon in all cases. Postoperative specimens were reviewed by consultant histopathologists for confirmation of diagnosis and evaluation of histological features, including lymphovascular invasion (LVI), extrathyroidal extension (ETE), and tumour size.

Statistical analysis

Statistical analysis was performed using IBM SPSS Statistics version 24 (IBM Corp., Armonk, NY, US). Continuous variables (e.g., age, TSH level, and tumour size) were reported as means and standard deviations, and comparisons between benign and malignant groups were assessed using independent samples t-tests. Categorical variables (e.g., sex, FNAC category, LVI, and ETE) were analysed using Pearson’s Chi-squared test. A p-value of <0.05 was considered statistically significant. Test statistics, including t-values and χ² values, were also calculated and reported alongside p-values to support the validity of the findings.

## Results

**Figure 1:**
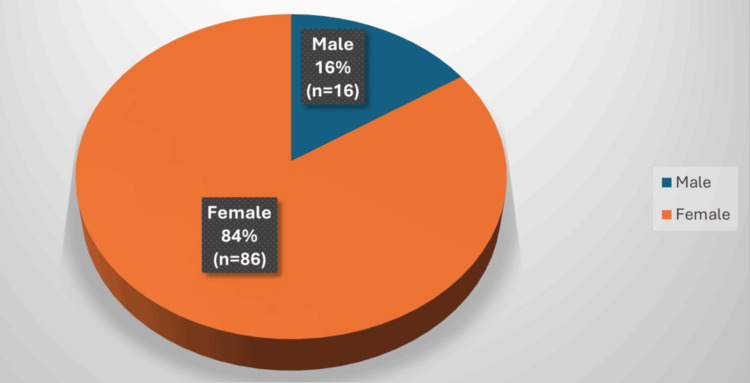
Sex Distribution of Patients

**Figure 2:**
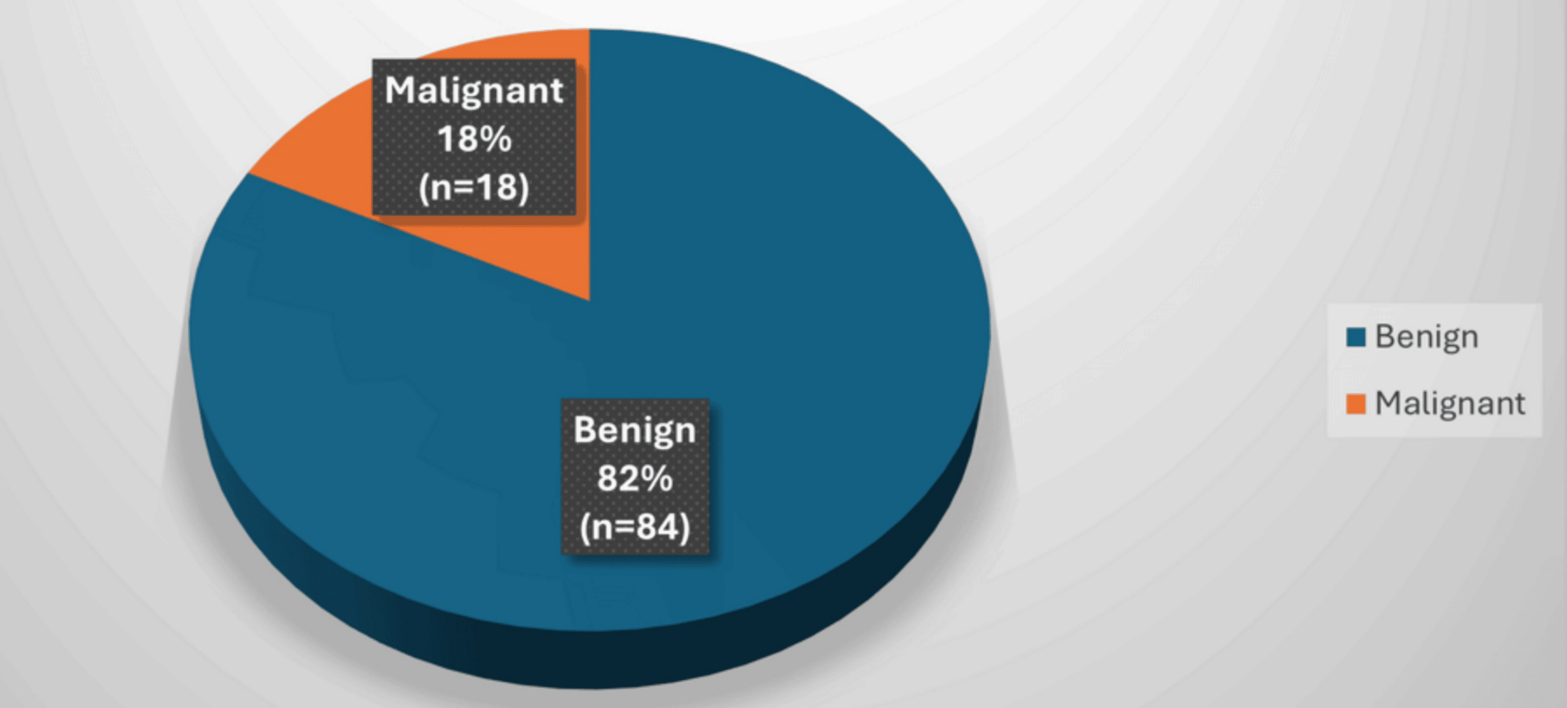
Malignancy Distribution in Thyroid Nodules

Among the 102 patients, 86 (84.3%) were women and 16 (15.6%) were men, with a mean age of 43.6 years. Malignant tumours were identified in 18 patients (17.6%), whilst 84 (82.4%) had benign lesions (Figures 1, 2). The most common malignancy was papillary thyroid carcinoma (PTC). Among the malignant cases, 15 were PTC, two were follicular carcinoma, and one was medullary carcinoma. No anaplastic carcinoma was observed.

Among the 84 benign cases, the most common histological diagnosis was multinodular goitre (n = 58), followed by colloid nodules (n = 19) and Hashimoto’s thyroiditis (n = 7). The predominance of multinodular goitre is consistent with its high prevalence in patients presenting with large, palpable thyroid nodules and its generally lower association with malignancy.

The mean sTSH level in the malignant group was 1.378 μIU/mL, compared to 1.350 μIU/mL in the benign group. Statistical analysis revealed no significant difference (p = 0.68). However, histopathological characteristics such as LVI and ETE were significantly more frequent in malignant cases (p < 0.01).

Out of the 102 patients, 77 (75.5%) underwent total thyroidectomy, whilst 25 (24.5%) had partial thyroidectomy. All malignant cases were treated with total thyroidectomy. The decision to perform total versus partial thyroidectomy was based on clinical and radiological features, including the presence of bilateral nodules, FNAC category, and ultrasound findings. Total thyroidectomy was performed in cases with Bethesda V cytology, bilateral disease, or high suspicion of malignancy. Partial thyroidectomy was considered for solitary, benign-appearing nodules or indeterminate lesions without concerning features. All surgical decisions were made by the consultant surgeon based on standard clinical criteria.

FNAC results were available for all patients. Of the 18 malignant cases, 11 (61.1%) had cytology categorised as ‘suspicious for malignancy’ or ‘malignant’, whilst seven cases (38.9%) were ‘indeterminate’ or ‘benign’ on FNAC, indicating limitations in preoperative cytological assessment. FNAC results were categorised using the Bethesda system. Although precise subclassification into Bethesda categories III, IV, and V was limited in some records, it was noted that a proportion of malignant cases fell into the indeterminate or benign categories preoperatively. Among cases classified as ‘suspicious for malignancy’, the majority were confirmed malignant on histology, supporting the known predictive value of higher Bethesda categories. No Bethesda VI cases were reported. These findings highlight the diagnostic limitations of FNAC in indeterminate lesions.

Ultrasound imaging data revealed that hypoechogenicity and irregular margins were more commonly associated with malignancy; however, a detailed quantitative analysis of these findings was not performed in this study. In terms of histological findings, ETE was observed in eight of 18 malignant cases (44.4%), whilst LVI was noted in seven cases (38.9%). These features were absent or minimal in benign lesions (p < 0.01). Tumour size averaged 91.4 mm in malignant versus 79.5 mm in benign lesions (p = 0.21). The patient demographics and tumour characteristics are summarised in Table 1. 

**Table 1:** Patient Demographics and Tumour Characteristics sTSH: serum thyroid-stimulating hormone; LVI: lymphovascular invasion; ETE: extrathyroidal extension; FNAC: fine-needle aspiration cytology t: independent samples t-test; χ²: Pearson's Chi-squared test

Variable	Benign (n = 84)	Malignant (n = 18)	Test statistic	p-value
Age (years)	43.2 ± 11.5	44.9 ± 12.1	t = 0.50	0.62
Female (%)	85.70%	83.30%	χ² = 0.06	0.79
Tumour size (mm)	79.5 ± 32.1	91.4 ± 35.7	t = 1.28	0.21
Mean sTSH (μIU/mL)	1.350 ± 0.92	1.378 ± 1.15	t = 0.41	0.68
LVI present (%)	2.30%	38.90%	χ² = 19.71	<0.01
ETE present (%)	3.50%	44.40%	χ² = 22.10	<0.01
FNAC malignancy (%)	4.70%	61.10%	χ² = 33.79	<0.01

## Discussion

Previous studies have extensively examined the relationship between TSH levels and thyroid malignancy risk, producing varied results. A retrospective analysis by Fiore et al. observed that higher TSH values were significantly associated with an increased risk of thyroid cancer [7]. Similarly, a large-scale investigation by Shi et al. analysed the clinicopathological risk and prognosis of papillary thyroid cancer variants and emphasised the potential role of hormonal regulation in disease progression [8]. These studies reinforce the importance of evaluating sTSH levels as part of a comprehensive preoperative work-up.

Several studies have suggested a potential relationship between higher TSH levels and increased malignancy risk. Recent meta-analyses, including the work by Wang et al., have confirmed a dose-response relationship between elevated thyroid hormone levels and increased thyroid cancer risk, supporting the hormone-neoplasia hypothesis [9]. Additional research has expanded on this relationship by demonstrating a dose-dependent risk increase with rising TSH levels. These large-scale evaluations support the hypothesis that elevated TSH is not merely an epiphenomenon but could actively participate in the progression of thyroid neoplasia [10]. Similarly, Fiore et al. reported that patients with TSH > 2.0 μIU/mL had a higher likelihood of developing PTC [7].

A nested case-control study by Huang et al. demonstrated that higher sTSH levels were significantly associated with an increased risk of papillary thyroid cancer, reinforcing the hormonal influence on thyroid tumourigenesis [11]. Su et al. conducted a systematic review and meta-analysis that confirmed a significant association between elevated preoperative TSH levels and thyroid cancer risk, suggesting that TSH could serve as a reliable preoperative marker [12]. These findings highlight the relevance of TSH in risk stratification for thyroid cancer.

In addition to TSH analysis, our study explored other predictive markers for malignancy. FNAC demonstrated high sensitivity, identifying malignancy in 61.1% of cases. However, a notable 38.9% of malignant cases were initially reported as indeterminate or benign on cytology, emphasising the diagnostic limitations of FNAC. Imaging findings, particularly hypoechogenicity and irregular margins, were more prevalent in malignant nodules, in line with previous studies by Rosario and Moon et al., which showed these features to have strong predictive value [13,14]. Furthermore, histological factors such as LVI and ETE were significantly associated with malignancy in our cohort, aligning with risk stratification models used in guidelines by the American Thyroid Association [15]. Whilst LVI and ETE are postoperative findings and not applicable to preoperative risk stratification, their inclusion provides insight into the biological behaviour and aggressiveness of malignant nodules. Although this study did not specifically evaluate ultrasound features such as nodule borders or echogenicity, previous literature has shown that nodules larger than 4 cm and those with irregular or heterogeneous ultrasound patterns may have higher false-negative rates on FNAC. Incorporating detailed ultrasound criteria in future studies would help refine preoperative risk stratification and improve diagnostic accuracy [16].

Collectively, our findings suggest that whilst sTSH alone may not be a reliable standalone predictor, it retains potential as part of a multimodal risk stratification approach alongside imaging and histopathology.

## Conclusions

This retrospective study analysed 102 patients undergoing thyroidectomy to evaluate the potential role of preoperative sTSH levels in predicting thyroid malignancy. Whilst a possible association between elevated sTSH and increased malignancy risk was suggested, our results demonstrated no statistically significant difference in TSH levels between benign and malignant cases. However, other clinical and histopathological factors-such as ETE, LVI, and ultrasound features-showed a strong association with malignancy. Our findings reaffirm the importance of multimodal risk stratification that integrates imaging, cytology, and histology in thyroid nodule evaluation.

It is important to acknowledge limitations including the relatively small sample size, retrospective nature of the analysis, and lack of data on confounding factors like iodine status or autoimmune thyroid disease. These factors may have influenced TSH values and malignancy risk. Future studies should focus on larger, multicentre prospective cohorts, incorporating molecular markers and long-term follow-up to better define the predictive value of TSH. Until such data become available, clinicians should continue to rely on established diagnostic methods, using sTSH as a supplemental-but not standalone-tool in the comprehensive assessment of thyroid nodules.
